# Potential biomarkers and immune characteristics for polycythemia vera-related atherosclerosis using bulk RNA and single-cell RNA datasets: a combined comprehensive bioinformatics and machine learning analysis

**DOI:** 10.3389/fcvm.2024.1426278

**Published:** 2024-08-12

**Authors:** Ziqing Wang, Jixuan Zou

**Affiliations:** ^1^ Beijing Friendship Hospital, Capital Medical University, Beijing, China; ^2^ Graduate School, Beijing University of Chinese Medicine, Beijing, China

**Keywords:** atherosclerosis, biomarkers, immune, polycythemia vera, machine learning analysis

## Abstract

**Background:**

Polycythemia vera (PV) is a myeloproliferative disease characterized by significantly higher hemoglobin levels and positivity for JAK2 mutation. Thrombosis is the main risk event of this disease. Atherosclerosis (AS) can markedly increase the risk of arterial thrombosis in patients with PV. The objectives of our study were to identify potential biomarkers for PV-related AS and to explore the molecular biological association between PV and AS.

**Methods:**

We extracted microarray datasets from the Gene Expression Omnibus (GEO) dataset for PV and AS. Common differentially expressed genes (CGs) were identified by differential expression analysis. Functional enrichment and protein-protein interaction (PPI) networks were constructed from the CG by random forest models using LASSO regression to identify pathogenic genes and their underlying processes in PV-related AS. The expression of potential biomarkers was validated using an external dataset. A diagnostic nomogram was constructed based on potential biomarkers to predict PV-related AS, and its diagnostic performance was assessed using ROC, calibration, and decision curve analyses. Subsequently, we used single-cell gene set enrichment analysis (GSEA) to analyze the immune signaling pathways associated with potential biomarkers. We also performed immune infiltration analysis of AS with “CIBERSORT” and calculated Pearson's correlation coefficients for potential biomarkers and infiltrating immune cells. Finally, we observed the expression of potential biomarkers in immune cells based on the single-cell RNA dataset.

**Results:**

Fifty-two CGs were identified based on the intersection between up-regulated and down-regulated genes in PV and AS. Most biological processes associated with CGs were cytokines and factors associated with chemotaxis of immune cells. The PPI analysis identified ten hub genes, and of these, CCR1 and MMP9 were selected as potential biomarkers with which to construct a diagnostic model using machine learning methods and external dataset validation. These biomarkers could regulate Toll-like signaling, NOD-like signaling, and chemokine signaling pathways associated with AS. Finally, we determined that these potential biomarkers had a strong correlation with macrophage M0 infiltration. Further, the potential biomarkers were highly expressed in macrophages from patients with AS.

**Conclusion:**

We identified two CGs (CCR1 and MMP9) as potential biomarkers for PV-related AS and established a diagnostic model based on them. These results may provide insight for future experimental studies for the diagnosis and treatment of PV-related AS.

## 1 Introduction

Polycythemia vera (PV) is a myeloproliferative disorder characterized by significantly increased hemoglobin levels and JAK2 gene mutation. PV is the most common myeloproliferative neoplasm, with an incidence of 10.9 per million. It is often accompanied by hematological features such as increased white blood cell (WBC) and platelet (PLT) counts, and clinical manifestations such as thrombosis, headache, gastrointestinal ulcer, pulmonary hypertension, and splenomegaly (1–3). Thrombosis is the main risk event for this disease, with an incidence of approximately 46%, and arterial thrombosis occurs 2–3 times more frequently than venous thrombosis (4, 5). Arterial thrombosis-related cardiovascular and cerebrovascular disorders pose a major risk to patient safety and quality of life, as well as an economic and public health burden to society. Atherosclerosis (AS) can significantly increase the risk of arterial thromboembolism in patients with PV, according to recent research (6). To prevent and reduce the incidence of thrombosis in patients with PV, it is crucial to evaluate the impact of AS in these individuals.

Patients with PV have a higher risk of AS and plays a significant role in arterial thrombosis in patients with PV. The growth and rupture of an atherosclerotic plaque is frequently a prerequisite for arterial thrombosis, acting as a prepathological stage of the disease. Arterial thrombosis may result from JAK2 mutation-induced leukocytosis and immunological dysfunction (7–9). Furthermore, the expression of cytokines by bone marrow cells in patients with PV can interfere with endothelial system homeostasis and further supports the potential that endothelial damage in patients with PV leads to AS (10–12). Although numerous studies have shown an intricate connection between PV and AS, additional studies are required to fully understand the implications and underlying biological processes between the two diseases (13–15).

**Figure 1:**
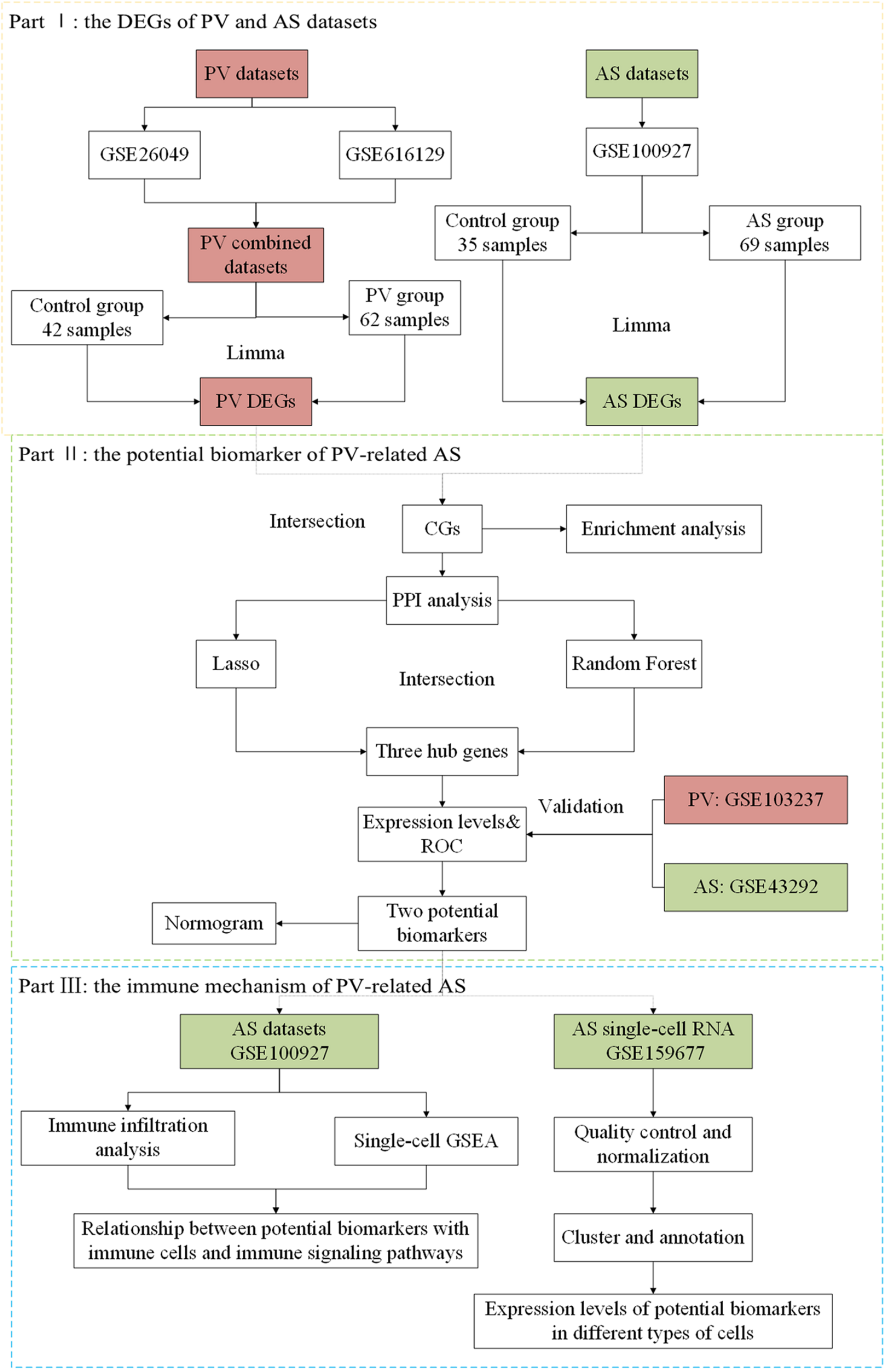
Flow chart of this study design.

Our hypothesis was that the pathogenic mechanisms underlying PV-related AS could be unraveled at the molecular biological level by employing bulk-RNA and single-cell RNA datasets and bioinformatics approach to thoroughly investigate abnormally expressed genes of the two diseases. In this study, we first explored the potential cellular and molecular pathways involved and thoroughly analyzed the association between PV and AS based on bulk-RNA and single-cell RNA datasets using various bioinformatics tools. We used a variety of advanced statistical algorithms to identify potential biomarkers of PV and AS and assessed their interaction and infiltrating immune cells. Additionally, the latent value of potential biomarkers in the diagnosis of disease was evaluated and validated in different cohorts (Figure 1).

## 2 Materials and methods

### 2.1 Data collection

Six human gene expression profile datasets were obtained from the GEO databases (https://www.ncbi.nlm.nih.gov/geo/) (16). Bulk transcriptome data of controls and patients with PV were contributed by GSE26049 (whole blood from 41 PV individuals and 21 control individuals), GSE61629 (whole blood from 21 PV individuals and 21 control individuals), and GSE103237 (bone marrow CD34 + cells from 21 PV individuals and 21 control individuals). Bulk transcriptome data of the control and AS patients was contributed by GSE100927 (35 control arteries and 69 atherosclerotic lesions) and GSE43292 (32 control arteries and 32 atherosclerotic lesions). Single-cell transcriptome data of AS patients were extracted from GSE159677 (3 AS patients).

### 2.2 Analysis of differentially expressed genes

Integrated PV expression data was obtained by the batch correction of GSE26049 and GSE61629 based on the “SVA” package (17) in R software (version 4.2.1), which contained 62 PV samples and 42 control samples. Gene symbol conversion, background correction, and normalization were performed on the integrated PV and the AS data sets (GSE100927). Subsequently, the differentially expressed genes (DEGs) in the PV and AS datasets were identified with the “Limma” package (18) in R software. The DEGs were filtered with a fold change ≥1.5 and p.adjust <0.05. Next, DEGs were summarized as volcano plots and heatmaps with the “ggplot2” and “pheatmap” packages in R software. As a result, the up- and down-regulated genes of PV and AS were intersected with the “dplyr” package in R software to identify CG.

### 2.3 Enrichment analysis of CGs from patients with AS and PV

Gene Ontology (GO) analysis and Kyoto Encyclopedia of Genes, Genomes (KEGG) analyses were performed to identify possible functions for the CGs from patients with AS and PV by “clusterProfiler” packages (19) in the R software. A *P*-value <0.05 was considered a significant statistical difference in the GO and KEGG analysis. Visualizations of GO and KEGG analysis were depicted with bar plot and chord diagrams by “ggplot2” package in the R software.

### 2.4 Construction of a protein-protein interaction network

To identify interactions among the CGs from patients with AS and PV, a protein-protein interaction (PPI) network was established on the basis of STRING data (https://www.string-db.org) (20), with a medium confidence score of >0.4. Later, the PPI network was visualized using Cytoscape software (version 3.8.0). We also used the Cytoscape plug-in molecular complex detection (MCODE) to detect the significant modules. The CGs in the modules with the highest scores were chosen for further analysis.

### 2.5 Machine learning

The least absolute shrinkage and selection operator (LASSO) method was used to screen for possible biomarkers using the “glmnet” package (21) in R software. Subsequently, the random forest (RF) modelling was used to further refine potential biomarkers using the “randomForest” package (22) in R software. The overlapping genes from the LASSO model and the RF model (MeanDecreaseGini >2) were defined as hub genes for the establishment of a diagnostic model of PV-related AS.

### 2.6 Expression of hub genes and evaluation of receiver operating characteristic

The expression patterns of the hub gene were first evaluated in the GSE100927 and integrated PV datasets, and then confirmed in the GSE103237 and GSE43292 datasets. The Wilcoxon test was used for comparison with a significance level of *P* < 0.05. ROC curves were produced to evaluate the diagnostic value of the hub gene for the diagnosis of PV and AS, respectively. AUC values >0.7 suggested a substantial difference. The AUC values and their accompanying 95% confidence intervals were calculated to separate the illness group from the control group.

### 2.7 Nomogram creation and the potential marker prediction model evaluation

The nomogram was created based on the hub genes using the “rms” package in R software. The performance of each hub gene and the nomogram in the diagnosis of AS was assessed by the area under the ROC curve. Furthermore, the ROC curve analysis was performed to assess the suitability of the nomogram-based diagnosis of AS. Lastly, the prediction efficiency of nomograms in PV-related AS was evaluated with calibration curves and decision curve analysis (DCA).

### 2.8 Implementation of gene set enrichment analysis (GSEA) for potential biomarkers

After acquiring potential biomarkers, we used the “clusterProfiler” (23) program to perform single-gene gene set enrichment analysis (GSEA) for every potential biomarker in AS datasets. Our objective was to analyze the signaling pathways regulated by the potential biomarkers in AS with GSEA. The MSigDB (c5.go.bp.v7.5.1.entrez.gmt) was used to obtain gene sets. A *P*-value < 0.05 was considered statistically significant. The immunological pathways for every gene in the AS datasets were represented using the “enrichplot” package in R software.

### 2.9 Immune infiltration analysis

The AS gene expression profile was used to determine the amount of immune cell infiltration based on “CIBERSORT” program (24). A bar plot representing the amount and percentage of immune infiltration was displayed for every sample with the “ggplot2” package. The proportions of 22 different immune cell types in the calcified and control aortic valve samples were compared using “kruskal-test”. A *P*-value < 0.05 was considered statistically significant, and the results were displayed in a stacked histogram created with the “ggplot2” software. Finally, the association between the expression of potential biomarkers and the amount of immune cells infiltration was then analyzed using the Pearson's rank correlation coefficient, with *P* < 0.05 regarded as statistically significant. A coefficient of correlation >0.7 was considered a strong correlative.

### 2.10 Single-cell RNA analysis

We used the “Seurat” and “SingleR” packages (25) to analyze AS single-cell RNA datasets. To preserve high-quality data, we excluded cells with a mitochondrial gene percentage >5%, cell counts <3, and cells expressing <300 and >5,000 genes. We used the “NormalizeData” function to normalize the gene expression of the included cells. Principal component analysis (PCA) was used to extract the top 20 principal components (PC) based on the top 2,000 highly variable genes. Finally, the “FindVariableFeatures” function was used to save these PCs for additional analysis. The “FindNeighbors”, “FindClusters” (resolution = 0.6), and “RunUMAP” programs were used to cluster cell subpopulations in an unsupervised and unbiased manner. The marker genes for each cluster were screened using the “FindAllMarkers” function with fold change ≥1.2 and p.adjust <0.05. Lastly, the “SingleR” package annotated cell types. Finally, “Featureplot” function was used to show the expression of potential biomarkers in the type of cells in AS.

## 3 Results

### 3.1 Identification of CGs in PV and AS datasets

**Figure 2:**
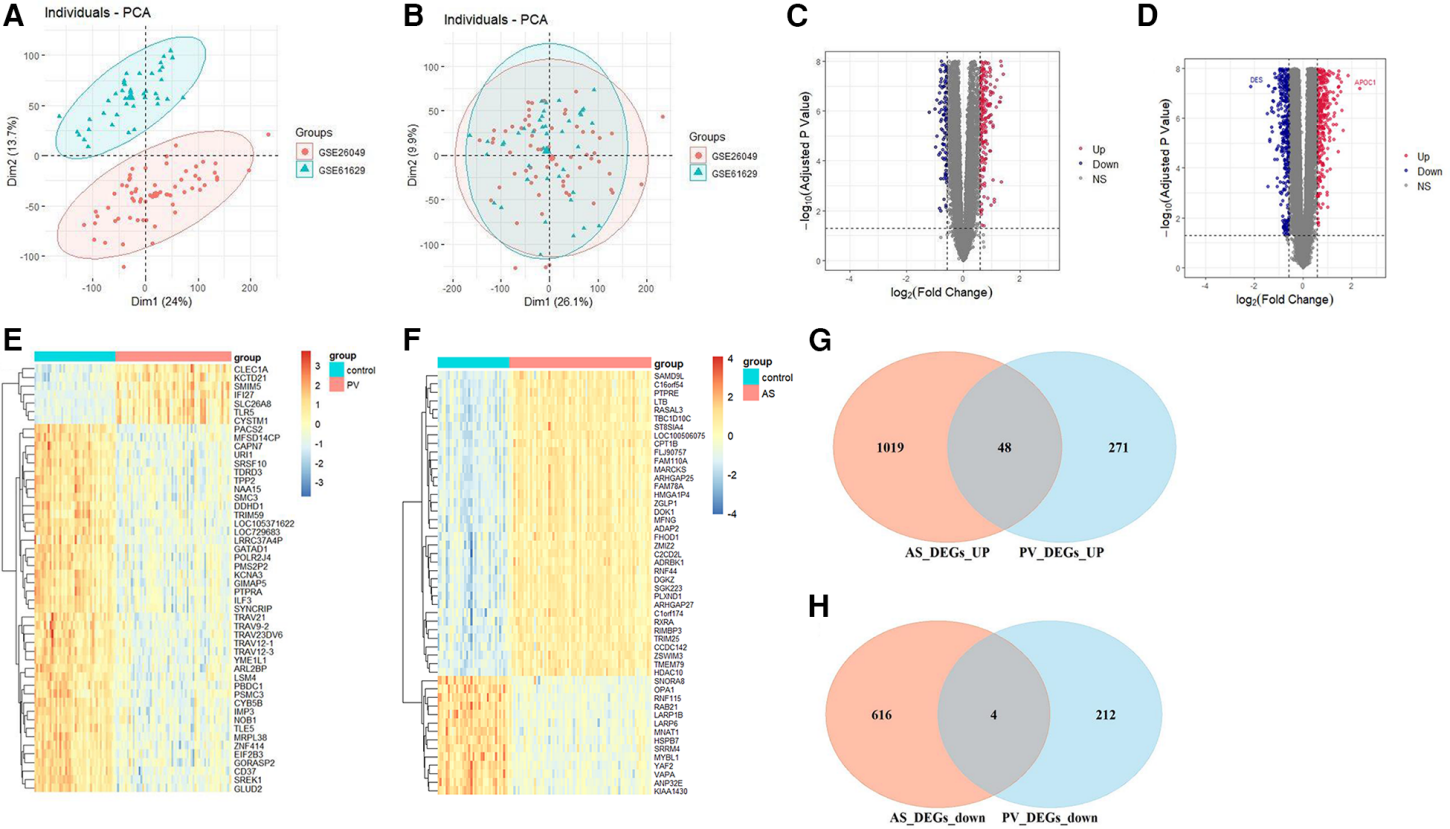
Differential expression gene analysis. PCA of two original PV datasets (**A)** before and (**B)** after batch-effect correction. The volcano plots of differentially expressed genes (DEGs) in the (**C**) PV and (**D)** AS datasets. The expression pattern for the top 50 CGs in the (**E)** PV and (**F**) AS datasets. Venn plots of (**G)** upregulated and **(H)** downregulated CGs between PV and AS datasets.

GSE26049 and GSE61629 were combated after batch correction (Figures 2A,B), the differences between two data sets were significantly decreased after batch effect removal. In the PV datasets (GSE26049 and GSE61629), a total of 535 DEGs, consisting of 319 upregulated DEGs and 216 downregulated DEGs, were identified (Figure 2C). In the AS data set GSE100927, a total of 1,687 DEGs, consisting of 1,067 upregulated DEGs and 620 downregulated DEGs, were identified (Figure 2D). The heatmaps show the top 50 expression pattern of CGs in PV and AS cohorts (Figures 2E,F). As shown in Figures 2G,H, there were 48 overlapping upregulated CGs and 4 overlapping downregulated CGs between PV and AS cohorts.

### 3.2 Biological function and pathways of identified CGs

**Figure 3:**
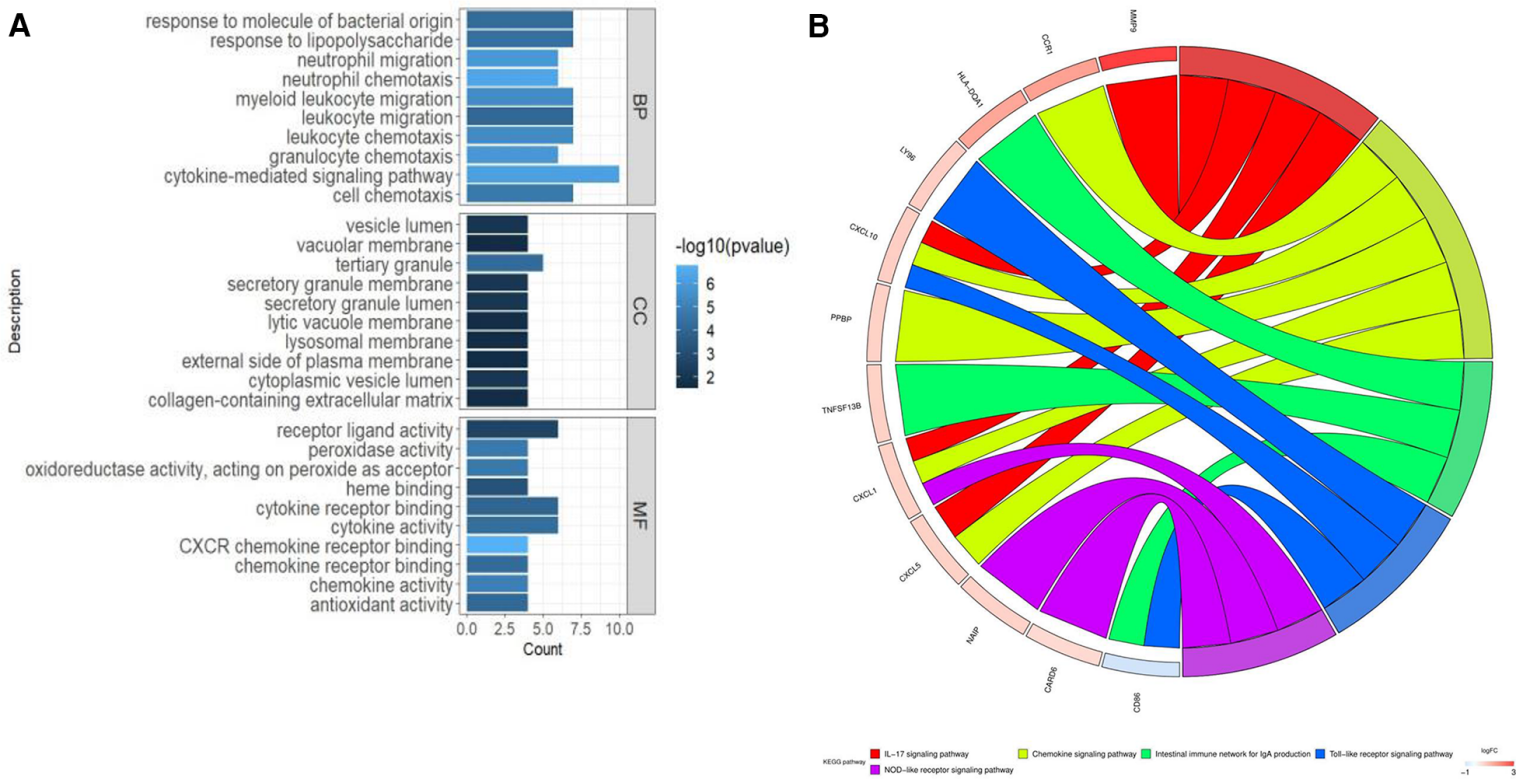
Enhanced function and pathway enrichment. (**A**) GO enrichment and (**B**) KEGG enrichment of CGs.

The biological role of CGs was examined using GO and KEGG enrichment analysis. The enriched biological processes (BPs) of CGs included neutrophil migration, neutrophil chemotaxis, myeloid leukocyte migration, leukocyte migration, leukocyte chemotaxis, granulocyte chemotaxis, and cytokine-mediated signaling pathways, and cell chemotaxis (Figure 3A). The enriched molecular function (MF) of CGs included receptor ligand activity, peroxidase activity, oxidoreductase activity, acting on peroxide as acceptor, heme binding, cytokine receptor binding, cytokine activity, CXCR chemokine receptor binding, chemokine receptor binding, chemokine activity, and antioxidant activity (Figure 3A). The enriched cell component (CC) of CGs included the vesicle lumen, vacuolar membrane, tertiary granule, secretory granule membrane, secretory granule lumen, lytic vacuole membrane, lysosomal membrane, and external side of plasma membrane (Figure 3A). The immunological and inflammatory pathways in which CGs were the most enriched were chemokine signaling, IL-17, Toll-like receptor, and NOD-like receptor signaling pathways, and the intestinal immune network for IgA production (Figure 3B).

### 3.3 Identification of optimal diagnostic value CGs

**Figure 4:**
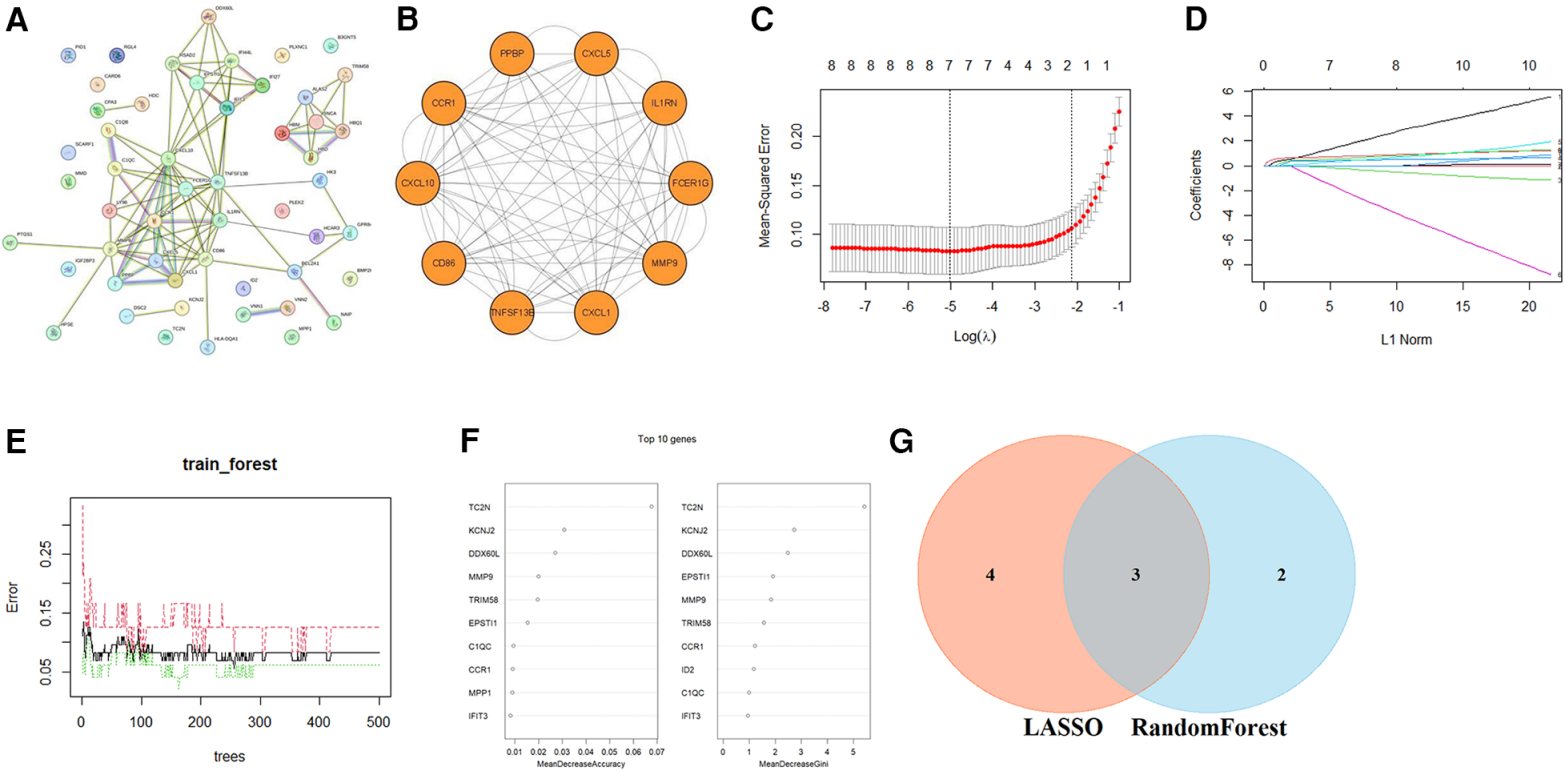
Identification of optimal diagnostic value CGs. (**A**) PPI network of CGs. (**B**) The most important modules in the PPI network of CGs. The minimum values (**C**) and lambda values (**D**) of potential biomarkers were identified by the LASSO logistic regression algorithm. (**E**) The error in AS shown by random forests (RF) survival algorithm. (**F**) RF algorithm that presents the MeanDecreaseGini of CGs in AS and biomarkers with the score >2.0 were selected. (**G)** Venn diagram displaying two common genes between the LASSO and RF algorithms, which were identified as the hub genes in PV-related AS.

To reveal the potential pathogenic genes and underlying mechanisms in PV-related AS, the interaction of the key genes in PV and AS was collected by the STRING database to identify probable pathogenic genes and the underlying mechanism in PV-related AS. The most important modules were identified through MCODE, which included the 10 genes that have been recognized as pathogenic genes in PV-related AS. (Figures 4A,B). Subsequently, seven possible candidate genes among ten CG were identified with the LASSO regression algorithm, which had a significant impact on the diagnosis of patients with PV who presented AS (Figures 4C,D). Five CGs were retrieved using the RF machine learning technique, which was also used to rank the ten CGs according to the varying relevance of each gene to further narrow the potential biomarkers (Figures 4E,F). Three potential biomarkers overlapped in both groups after superposing the seven candidate genes from LASSO and the five probable genes from RF modelling, including motif chemokine receptor 1 (CCR1), C-X-C motif chemokine ligand 5 (CXCL5), and matrix metalloproteinase 9 (MMP9); all potential biomarkers were all up-regulated (Figure 4G).

**Figure 5:**
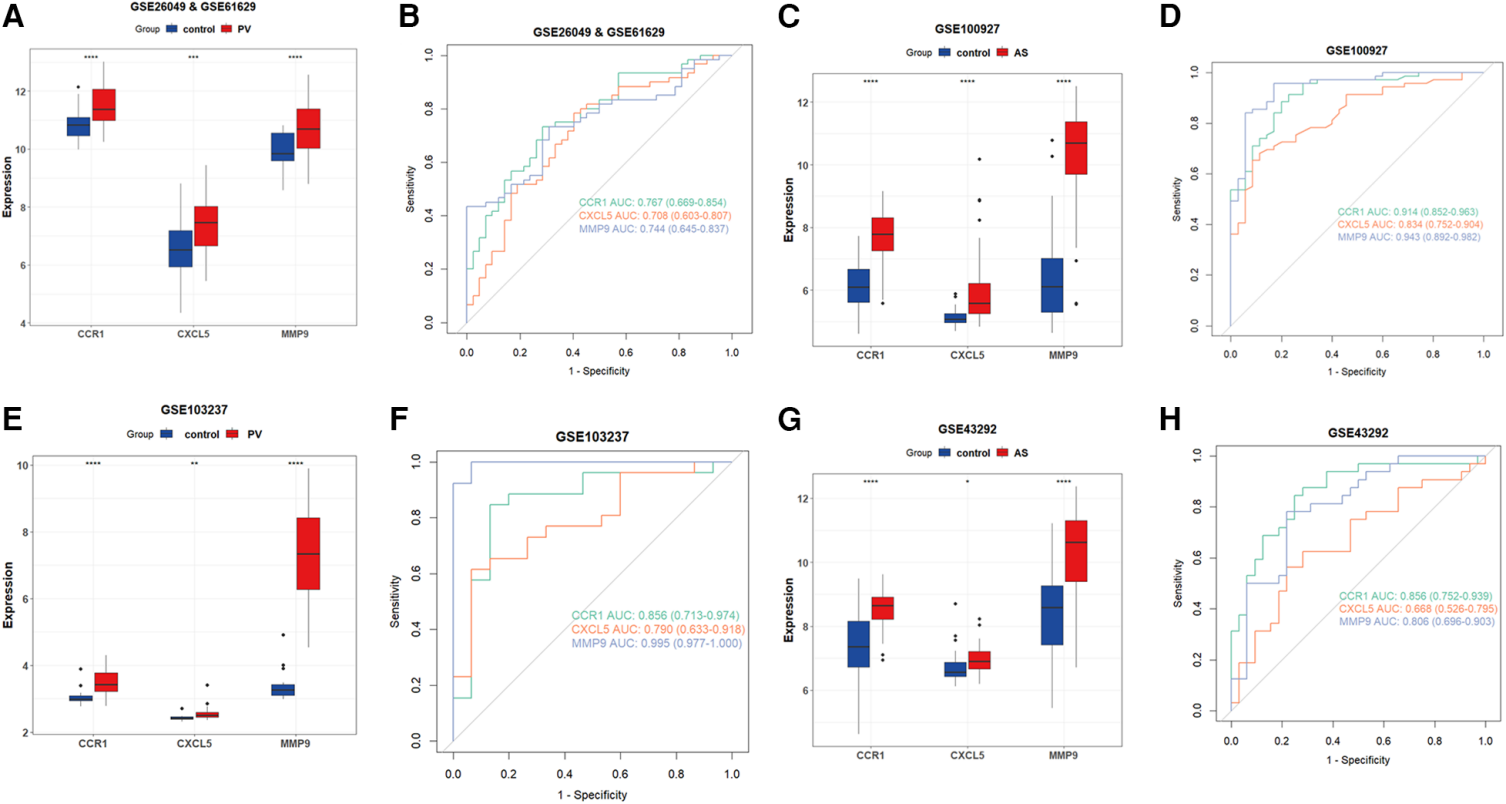
Verification of CCR1, CXCL5, and MMP9 as hub genes. (**A**) Expression of potential biomarkers in the GSE26049 and GSE61629 datasets. (**B**) ROC curve for potential biomarkers in the combined GSE26049 and GSE61629 datasets. (**C**) The expression level of potential biomarkers in the GSE100927 dataset. (**D**) ROC curve for potential biomarkers in the GSE100927 dataset. (**E**) The expression level of potential biomarkers in the GSE103237 dataset. (**F**) ROC curve for potential biomarkers in the GSE103237 dataset. (**G**) The expression level of d potential biomarkers in GSE43292 dataset. (**H**) ROC curve for potential biomarkers in the GSE43292 dataset. Statistical significance at the ns ≥ 0.05, * < 0.05, ** < 0.01, *** < 0.001, and **** < 0.0001 levels.

Subsequently, we examined the expression of potential biomarkers in patient groups from the AS dataset (GSE100927) and PV datasets (combined of GSE26049 and GSE61629) and compared them with control groups. The expression of potential biomarkers was significantly different between the patient and control groups (*P* < 0.05), and the ROC analysis supported the potential biomarkers as the most promising diagnostic marker for this condition (Figures 5A–D). Because the AUC of CXCL5 in GSE43292 was <0.7, CXCL5 was not suitable as a biomarker. Furthermore, the results of the external validation analysis demonstrated a significant difference in the expression of CCR1 and MMP9 (*P* < 0.05) between the patient and control groups (Figures 5E–H).

### 3.4 Construction and evaluation of a diagnostic model in PV-related AS

**Figure 6:**
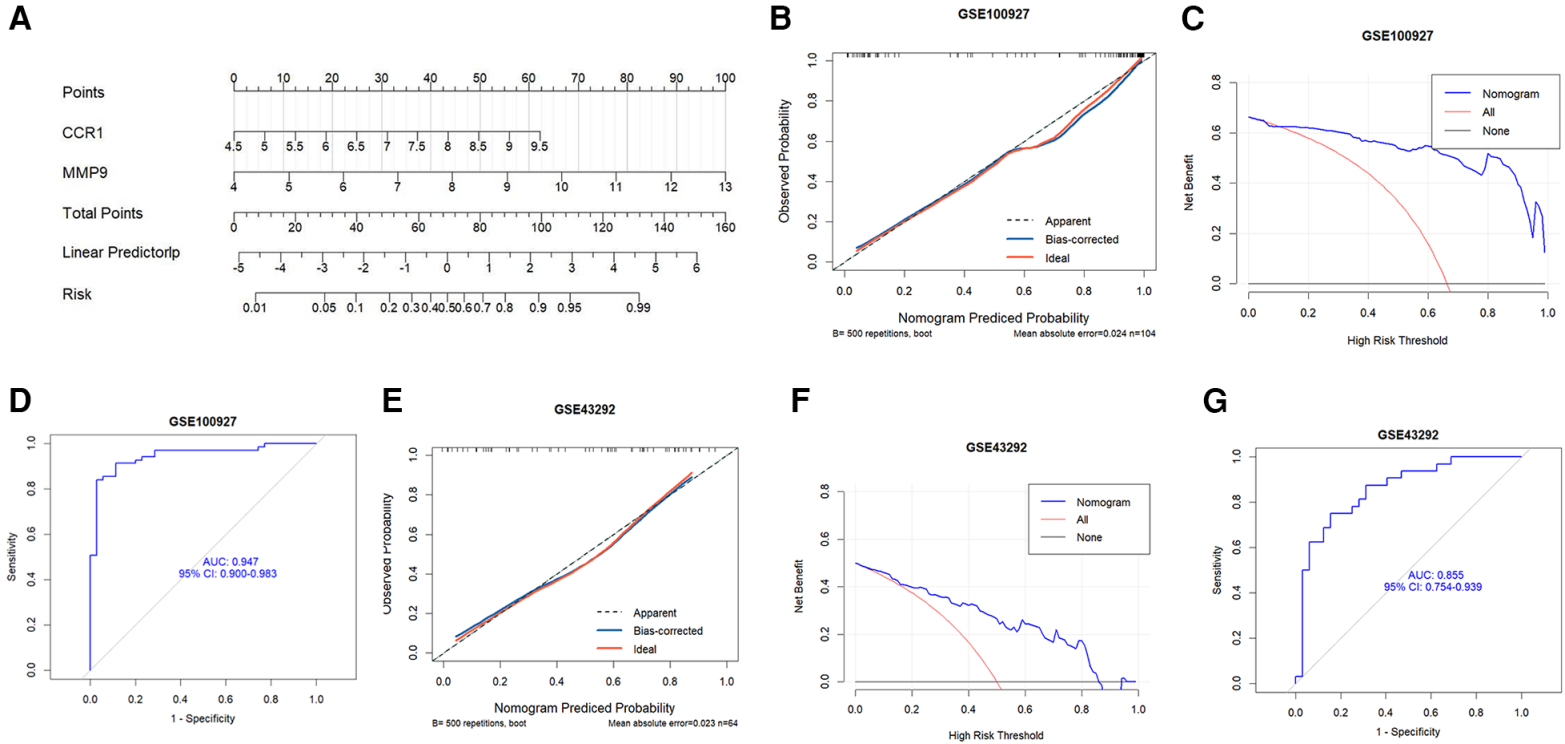
Development of the diagnostic nomogram model and evaluation of efficacy. (**A**) The nomogram was constructed on the basis of the potential biomarkers in PV-related AS. (**B**) The calibration curve of nomogram prediction model in the training dataset (GSE100927). (**C**) DCA for the nomogram model in the training dataset (GSE100927). (**D**) ROC curve for the diagnostic performance of the nomogram model in the training dataset (GSE100927). (**E**) Calibration curve for the nomogram prediction model using the validation dataset (GSE43292). (**F**) DCA for the nomogram model in the validation dataset (GSE43292). (**G**) The ROC curve evaluating the diagnostic performance of the nomogram model in the validation dataset (GSE43292).

A nomogram was created based on the two hub genes using logistics regression analysis to predict the possibility of developing AS in patients with PV (Figure 6A). The calibration curves revealed that the probability of the diagnostic nomogram model was approximately the same as that of the ideal model (Figure 6B). Furthermore, the DCA of the nomogram demonstrated that the nomogram model could be advantageous for the diagnosis of PV-related AS (Figure 6C). The nomogram had a high AUC value, indicating that it could be a useful diagnostic tool for AS-related PV (Figure 6D). Furthermore, the nomogram exhibited a great predictive value in the GEO GSE43292 dataset (Figures 6E–G), which means that the nomogram also had good predictive performance in the external cohorts.

### 3.5 Connection between potential biomarkers and inflammatory and immune processes in AS

**Figure 7:**
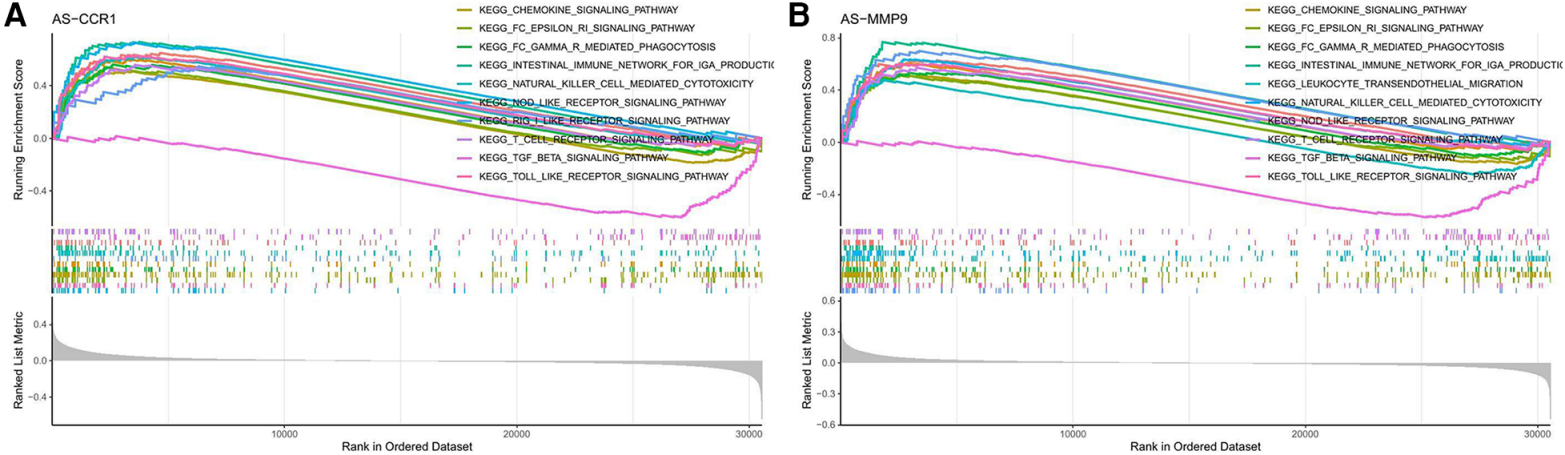
GSEA for potential biomarkers. GSEA analysis for (**A**) CCR1 and (**B**) MMP9 in the AS group.

Based on the GO and KEGG analysis of CGs in the PV and AS datasets, we found that CGs were closely associated with inflammatory and immune processes. Therefore, we used single-gene GSEA analysis of the two potential biomarkers in the AS group to determine the regulatory status of potential biomarkers for AS through immune pathways. CCR1 and MMP9 were both involved in immune pathways such as chemokine signaling, NOD-like receptor signaling, TOLL-like receptor signaling, FcεRI signaling, and in FCγR mediated phagocytosis (Figures 7A,B).

### 3.6 Immune cell infiltration and correlation analysis of potential biomarkers with invading immune cells in AS

**Figure 8:**
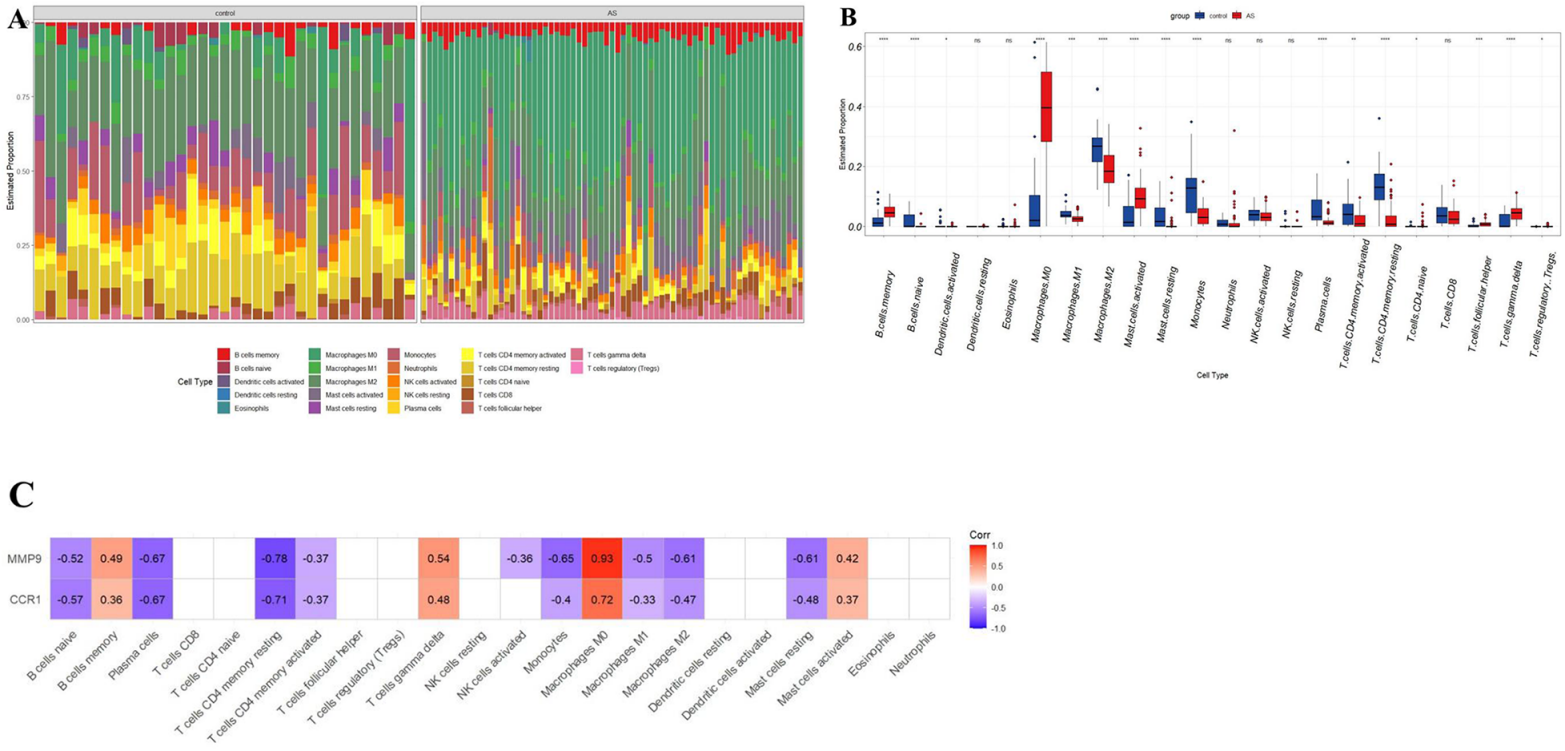
Immune cell infiltration analysis in AS. (**A**) Stacked histogram displaying the immune cell type proportions between AS and control groups. (**B**) Box plot showing the comparison of 22 immune cell types between AS and control groups. (**C**) The correlation map shows the association between two potential biomarkers and differentially infiltrated immune cells with a *P*-value < 0.05. AS, calcific aortic valve disease. *P < 0.05; **P < 0.01; ***P < 0.001; ****P < 0.0001; ns, not significant.

The potential biomarkers CCR1 and MMP9 showed a close association with inflammatory and immune processes in AS. Sequentially, the CIBERSORT algorithm was used to determine the properties of immune cells and to investigate the association between potential biomarkers and immune cell type in AS. Unlike the control group, AS exhibited higher proportions of macrophages M0, memory B-cells, activated mast cells, naive CD4T cells, follicular helper T cells, γδT cells, and regulatory T cells (Tregs), whereas lower proportions of naive B cells, activated dendritic cells, M1 macrophages, M2 macrophages, resting mast cells, monocytes, plasma cells, and active memory CD4T cells, and resting memory CD4T cells (Figures 8A,B). Furthermore, the expression of two potential biomarkers demonstrated a strong correlation with accumulation of M0 macrophages in AS (Figure 8C).

### 3.7 Expression of hub genes in single-cell RNA-seq datasets

**Figure 9:**
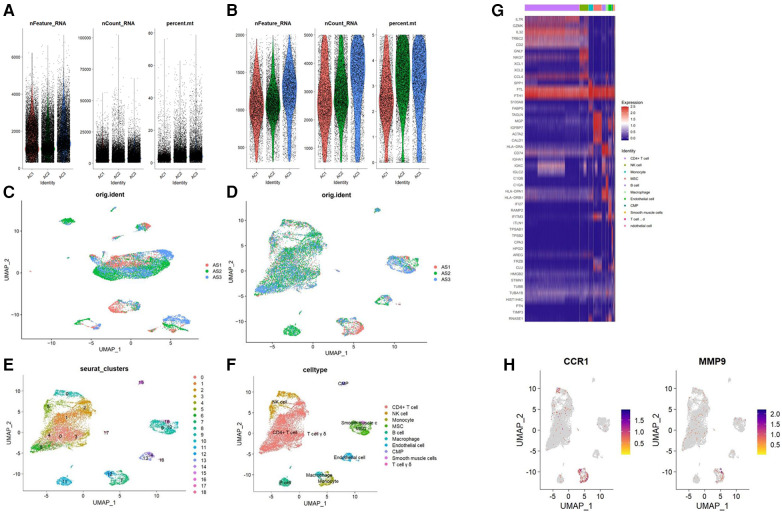
Analysis of single-cell RNA datasets. (**A**) Gene counts per cell (nFeature_RNA), the number of unique molecular identifiers (UMIs) per cell (nCount_RNA), and the proportion of mitochondrial genes per cell (percent.mt) of the data before the quality control. (**B**) nFeature_RNA, nCount_RNA, and the percent.mt of the data after quality control. UMAP plot of the data (**C**) before and (**D**) after normalization. (**E**) Cells were divided into 19 separate clusters. (**F**) Cells were clustered into 9 cell types. (**G**) Heatmap showing the expressions of the top 5 marker genes among 9 cell types. (**H**) Feature plots showing the distribution of potential biomarkers in various cell types.

The gene expression profiles of 38,611 cells from three AS samples were obtained from the GSE159677 dataset. We filtered the data according to the depth of sequencing and the number of genes found (Figures 9A,B), and normalized the data (Figures 9C,D). For further examination, the top 2,000 highly variable genes were chosen. The “RunPCA” function was used to reduce dimensionality and a total of 19 clusters were identified (Figure 9E). Subsequently, we annotated and visualized nine different cell types (CD4+ T cell, NK cell, monocyte, MSC, B cell, macrophage, endothelial cell, CMP, smooth muscle cells, and T cell yō) with the “SingleR” function (Figure 9F). The top five genes of every cell type validated the annotation using the “SingleR” function (Figure 9G). Unlike other immune cell types, we observed that hub genes were substantially expressed in macrophages when we examined the location and expression of CCR1 and MMP9 (Figure 9H).

## 4 Discussion

PV related to arterial thrombosis is a significant public health concern and economic burden and is a major risk factor and event for patients with PV. AS is a significant risk factor for arterial thrombosis in patients with PV (6). Furthermore, acquired alterations in immune cells are directly responsible for the worsening of atherosclerotic plaque inflammation, which in turn causes arterial thrombosis in patients with PV (7–9). Genes associated with atherosclerosis have also been confirmed to be highly dysregulated in MPNs (26). This study is the first to integrate bulk RNA and single-cell RNA data and to apply bioinformatics and machine learning methods to discover potential biomarkers for PV-associated AS and to explore the association between inflammation and immunity and PV-associated AS.

Our study identified 52 CGs from PV and AS datasets. Subsequently, we constructed a PPI network of CGs using the String database. Next, ten CGs were chosen using MCOD. Finally, three core CGs (CCR1, CXCL5, and MMP9) were obtained by combining the results of the LASSO regression and RF algorithms. CCR1 and MMP9 were finally identified via external data sets as core potential biomarkers for PV-associated AS. We constructed the diagnosis model based on these two potential biomarkers. CCR1 and MMP9 are both important genes involved in inflammation and immunoreaction and have a close connection with PV and AS. CCR1 is an important leukocyte chemokine receptor for several ligands, including CCL3 and MIP-1α. A large increase in serum CCL3 expression has been reported in patients with PV, and a significant increase in CCR1 expression has been described in a study of the neutrophil transcriptome of these patients (27). CCR1 is expressed in AS-related cell types, such as T lymphocytes and monocytes/macrophages, and mediates CCL5-dependent inhibition and transepithelial infiltration (28, 29). MMP9 is a zinc-dependent endopeptidase that is crucial for leukocyte motility and local proteolysis of the extracellular matrix. Using an animal model, bone marrow-derived JAK2-V617F mutant mice macrophages had a noticeably higher level of MMP9 mRNA expression (30). According to a different clinical study, the polymorphism of the MMP9 gene was associated with PV and may have a significant role in the increase in the risk of thrombosis (31). MMP-9 controls cholesterol metabolism via the MMP-9-plasma secreted phospholipase A2 axis, and MMP expression is markedly up-regulated in regions where macrophages are concentrated (around the lipid-rich core) (32, 33). The MMP9 of macrophages is crucial for the formation of susceptible regions of the AS plaque.

According to our GO enrichment analysis of 52 CGs, immune cell migration and chemotaxis were the primary biological processes of PV and AS. According to the KEGG enrichment analysis, CGs were enriched primarily in Toll-like receptors, NOD-like receptors, and chemokine signaling pathways. With single-gene GSEA, potential biomarkers included Toll-like receptor signaling pathways, NOD-like receptor signaling pathways, and chemokine signaling pathways in AS.

Toll-like receptors (TLRS) are membrane-bound receptors that generate innate immune responses, and aberrant activation of TLR3 is common in inflammatory or autoimmune diseases. Studies have shown that TLR polymorphisms may be protective against PV disease. Furthermore, the JAK2-Akt signaling pathway contributes to the pathophysiology of AS and is involved in the activation of monocyte chemoattractant protein-1 (MCP-1), which is necessary for monocyte migration into blood arteries and is induced by TLR (34, 35). TLR3 up-regulates *P*-selectin and vascular cell adhesion molecule-1 (VCAM-1) in injured endothelial cells, facilitating leukocyte adherence (mostly lymphocytes and monocytes) in blood and endothelial cell infiltration (36). TLR may also exacerbate the inflammatory state of thrombosis by stimulating the PLT and monocyte-macrophage system (37–39). NOD-like receptors (NLRs) are a specific family of pattern recognition receptors that are responsible for the generation of innate immune responses and play a key role in the recognition of intracellular ligands. NLRP3 is highly expressed in bone marrow cells from MPN patients, and its increased expression is associated with the JAK2V617F mutation, WBC counts and splenomegaly (40). In advanced AS, the NLRP3 inflammasome can induce premature macrophage death and massive lipid release, thus increasing plaque susceptibility (41). Various chemokines can activate the JAK/STAT, Ras, ERK, and Akt pathways through their receptors to induce directional chemotaxis of immune cells. Chemokine levels (CCL2, CCL5, CXCL8, CXCL12, CXCL10) were elevated in the bone marrow of patients with PV, indicating a highly inflammatory environment (42). Chemokines and their receptors may play a key role in the pathogenesis of AS. Chemokine receptors such as CCR1, CCR5, and CXCR2 can promote plaque development by recruiting monocytes into the blood. Elevated CXCL10 concentration is associated with an increased risk of vulnerable plaque and atherothrombosis (43).

Additionally, our study observed a strong connection between potential biomarkers and the infiltration of M0 macrophages in AS. Potential biomarkers were predominantly expressed in macrophages in AS plaque tissue, according to single-cell annotation and tissue marker gene analysis. This suggests that one of the primary mechanisms of PV-associated AS may be macrophage infiltration. Peripheral blood from PV patients contains considerably higher levels of inflammatory CD14 and CD16 monocytes/macrophages in various studies (44, 45). Macrophages are crucial at every stage of AS. During the inflammatory phase of AS, injured endothelium cells emit chemokines that attract circulating monocytes, which then undergo macrophage differentiation in response to growth factors and pro-inflammatory cytokines. These macrophages can quickly identify oxidized LDL, phagocytose it, and transform into foam cells, which form the first atherosclerotic lesions (46). The late stage of AS is characterized by a progressive increase in M1 macrophage counts and an increase in the release of pro-inflammatory factors, which increases the risk of plaque rupture (47). Furthermore, M1 macrophages have the potential to release MMPS, including MMP2 and MMP9, which cause the extracellular matrix of plaques to degrade and eventually rupture, increasing the risk of acute cardiovascular events (48).

However, our study has several limitations that should be addressed. Firstly, our study was constructed using retrospective data from public databases. Some bias may occur due to the limited sample size and multiple data analyses. Therefore, data from real-world studies are needed to verify the model's clinical applicability. In the future, we plan to collect blood samples from patients with initial onset of disease to confirm the expression and probable function of CGs.

## 5 Conclusions

This study identified two CGs (CCR1 and MMP9) as potential biomarkers for PV-related AS and established a diagnostic model based on these genes. Our results showed that Toll-like receptor signaling, NOD-like receptor signaling, and chemokine signaling may be closely associated connection with PV-related AS. Macrophage infiltration may be one of the primary mechanisms underlying PV-related AS. This study describes two potential markers for assessing the risk of developing AS in patients with PV and provides a new perspective on the common molecular mechanisms underlying PV and AS, which may provide clues for designing experimental studies, determining diagnosis, and widening treatment options for PV-related AS.

## Data Availability

Publicly available datasets were analyzed in this study. This data can be found here: (https://www.ncbi.nlm.nih.gov/geo/) GSE26049 GSE61629 GSE100927 GSE43292 GSE103237 GSE159677.
